# The Effects of Maternal Smoking on Pregnancy and Offspring: Possible Role for EGF?

**DOI:** 10.3389/fcell.2021.680902

**Published:** 2021-08-16

**Authors:** Hamed Janbazacyabar, Marthe van Daal, Thea Leusink-Muis, Ingrid van Ark, Johan Garssen, Gert Folkerts, Jeroen van Bergenhenegouwen, Saskia Braber

**Affiliations:** ^1^Division of Pharmacology, Utrecht Institute for Pharmaceutical Sciences, Faculty of Science, Utrecht University, Utrecht, Netherlands; ^2^Danone Nutricia Research, Utrecht, Netherlands

**Keywords:** epidermal growth factor, pregnancy, airway hyper responsiveness, cigarette smoke, immune response, inflammation

## Abstract

Cigarette smoke exposure during pregnancy and lactation is associated with adverse pregnancy outcomes. Here, we investigated the effects of maternal smoke exposure on pregnancy and offspring immunity and explored whether, epidermal growth factor (EGF), an important growth-promoting factor in human colostrum and milk, might be a possible missing link in maternal smoke exposure and changes in infants’ immune responses. Pregnant BALB/c mice were exposed to either cigarette smoke or air during gestation and lactation, and effects on pulmonary inflammation in dams and immune responses in offspring were examined. Maternal smoke exposure increased airway hyperresponsiveness and accumulation of inflammatory cells in the lungs of pregnant dams compared to non-pregnant dams. The E-cadherin protein expression was reduced in mammary glands of cigarette smoke-exposed pregnant dams. EGF levels were higher in mammary glands and serum of smoke-exposed pregnant dams compared to air-exposed pregnant dams. Offspring from cigarette smoke-exposed dams exhibited elevated levels of IL-17A, MCP-1, IL-22, and IL-13 in anti-CD3 stimulated spleen cell culture supernatants. EGF levels were also increased in serum of offspring from smoke-exposed dams. A positive correlation was observed between serum EGF levels and neutrophil numbers in bronchoalveolar lavage fluid of the dams. Interestingly, IL-17A, MCP-1, IL-22, IL13, and IFN-γ levels in anti-CD3 stimulated spleen cell culture supernatants of male pups also showed a positive correlation with EGF serum levels. In summary, our results reveal that maternal smoke exposure predisposes dams to exacerbated airway inflammation and offspring to exacerbated immune responses and both phenomena are associated with elevated EGF concentrations.

## Introduction

Smoking cigarettes throughout pregnancy is one of the most important preventable causes of adverse pregnancy outcomes (Hofhuis et al., 2003; Rauschert et al., 2019). Despite the well documented adverse health effects of maternal smoking and many efforts to reduce its prevalence, 20–30% of women who smoke, continue smoking during pregnancy and lactation (Dietz et al., 2010; Napierala et al., 2016). Besides active smoking, passive smoking (also known as environmental tobacco exposure (ETS) or second hand smoking) has also been linked to several diseases, including asthma and cancer. Because studies in non-smokers have demonstrated that exposure to ETS is very common worldwide, ETS is also often used as a proxy for indoor air pollution (Gilmour et al., 2006). According to the World health Organization (WHO), over 80% of the world’s 1.3 billion smokers live in low- and middle- income countries, and around 1.2 million deaths per year are attributable to ETS, worldwide. In addition, epidemiological studies have demonstrated high rates of ETS among the youth in these low- and middle-income countries (Wang et al., 2017; Elf et al., 2018). ETS contains the same range of tobacco smoke toxins as active smoking, therefore it is likely that ETS during pregnancy may also have important effects on fetal health. Maternal smoke exposure during pregnancy and lactation adversely affects birth weight (Bernstein et al., 2005), delivery time, fetal growth (Kolås et al., 2000; Fantuzzi et al., 2007), and immune development (Noakes et al., 2006). Cigarette smoke (CS) is an extremely complex and dynamic mixture containing more than 4000 harmful chemical compounds, (Bluhm et al., 1971; Ding et al., 2008). Especially, the smaller particles (<1–5 μm) penetrate deeply into the lung tissue, crossing the lung barrier and reaching the blood circulation (Antonietta Gatti, 2015). Hence, in addition to the local pulmonary injury, CS has far-reaching consequences on systemic immune responses, directly causing alterations in the innate and adaptive host defense, such as elevated white blood cell counts, and increased C-reactive protein (CRP), interleukin (IL)-6, and fibrinogen levels (Bermudez et al., 2002; Sopori, 2002; Helmersson et al., 2005; Wannamethee et al., 2005; Mehta et al., 2008). Moreover, both *in vivo* and *in vitro* studies have shown that maternal smoking is associated with substantial changes in the placental morphology (Asmussen, 1977; Kawashima et al., 2014), which consequently lead to impairment of the placental barrier (Demir et al., 1994). Results even indicated that CS components with a low molecular weight and high water solubility can cross the placental barrier and cause fetal injury (Sabra et al., 2017).

In addition to the placental effect, cigarette smoking during pregnancy reduces basal prolactin levels leading to a decrease in milk supply (Greenberg et al., 1991; Primo et al., 2013), changes in milk composition and flavor (Hill and Aldag, 1996), and early weaning (Primo et al., 2013). Breast milk is a unique source of nutrition containing various macronutrients (carbohydrates, proteins, lipids, and vitamins), as well as numerous bioactive compounds (growth factors, hormones, cytokines, chemokines, and antimicrobial compounds) for newborn infants. Epidermal growth factor (EGF) is an important growth-promoting factor in human colostrum and milk and is suggested to be responsible for the protective effects of milk on the gastrointestinal tract (GI) of newborn infants (Dvorak et al., 2003). EGF exposure starts in fetal life, as during pregnancy, EGF concentrations gradually increase in amniotic fluid reaching the highest concentration toward the end of gestation (Dvorak, 2010). After parturition and in the postnatal period, maternal colostrum and milk are the main source of EGF for the newborn offspring (Dvorak, 2010). The first days after parturition, the human EGF levels are high and gradually decrease during the first 2 weeks of lactation (Moran et al., 1983; Dvorak, 2010).

Several studies have linked smoke exposure during pregnancy and lactation to an increased risk of immune related diseases in the offspring. However, literature on how smoke exposure during pregnancy and lactation affects the infant’s immune system is scarce. The present study aims to investigate the effects of maternal CS-exposure on pregnancy and offspring immunity and to explore whether EGF might be a possible missing link in maternal smoke exposure and changes in infants’ immune responses.

In this study, important lung parameters and EGF levels in serum and mammary gland were explored in pregnant and non-pregnant dams exposed to air or CS. In the offspring, sensitivity of splenic immune cells and serum EGF levels were investigated to determine whether correlations can be found between EGF and changes in the offspring’s immune system.

## Materials and Methods

### Animals

Sixty females and thirty males 8-week-old specific pathogen free BALB/c by JIco mice were obtained from Charles River Laboratories (Someren, Netherlands). Upon arrival, mice were conventionally housed in groups (Female: 6/cage; Male: 5/cage used for mating) in filter-topped makrolon cages (22 cm × 16 cm × 14 cm, floor area 350 cm^2^, Tecnilab- BMI, Someren, Netherlands) with wood-chip bedding (Tecnilab- BMI, Someren in Netherlands) and tissues (VWR, Netherlands) were available as cage enrichment at the animal facility of Utrecht University. Animals were housed under a light/dark cycle of 12 h/12 h (lights on from 7.00 am to 7.00 pm) at controlled relative humidity (relative humidity of 50–55%) and temperature (21 ± 2°C) with *ad libitum* access to tap water and pelleted food (AIN-93G, Ssniff Spezialdiäten, Soest, Germany). After mating, cardboard houses were added to the cages. This study was conducted in accordance with institutional guidelines for the care and use of laboratory animals established by the Animal Ethics Committee of the Utrecht University, and all animal procedures related to the purpose of the research were approved under license of the national competent authority, securing full compliance the European Directive 2010/63/EU for the use of animals for scientific purposes.

### Mating, Gestation, and Smoke-Exposure

After the acclimatization period (2 weeks) and one day prior to mating, female animals were randomly housed two per cage and assigned to the control (*n* = 30) or cigarette smoke (CS) exposure group (*n* = 30). The next day, a single male mouse was introduced to two females [considered as pregnancy day (P) 0]. After 4 days of mating, male mice were removed from the female cages. Afterward, from P4 until the end of lactation, the females were subjected to whole body exposure (pups remained in the cage with extra tissues to maintain temperature) in whole-body chambers (8 animals/chamber) to either air or diluted mainstream CS from the reference cigarettes 1R6F (University of Kentucky, Lexington, Kentucky) using smoke apparatus. Exposures were performed 45 min/day (resembling 14 cigarettes/day), 7 days/week, for 6 weeks (P4 until the end of lactation) (Zuo et al., 2014). Carbon monoxide (CO) levels was monitored continuously and was approximately 300–400 ppm. The mass concentration of cigarette smoke total particulate matter (TPM) was determined by gravimetric analysis using a type A/E glass fiber filter (PALL life sciences, Mexico). Briefly, the glass filter was weighed and placed in the filter holder. Next, CS was passed through the filter. TPM was calculated by subtracting the basal filter weight from the CS-exposed filter divided by the volume of the filter holder and expressed as μg/L (Braber et al., 2012). The TPM concentration in the smoke exposure box generated by 14 cigarettes reached approximately 828 μg/L (828 ± 4.5 μg/L). At birth, the two mothers with their pups were maintained in filtered-top cages, and the numbers of viable offspring, along with the approximate duration of gestation, were determined. After 3 weeks of lactation, pups were weaned, sexed, and the sex ratio (male/female) and body weight were evaluated. After weaning, prenatally air and smoke-exposed pups were separately pooled and randomly assigned to the prenatal air or CS-exposure groups, analyzed for enhanced pause (Penh) and subsequently sacrificed (*N* = 10 pups/sex/exposure group). An overview of the experimental design is presented in Supplementary Figure 1.

### Airway Responsiveness Measurement

#### EMKA

The EMKA invasive measurement of dynamic resistance (EMKA Technologies, Paris, France) in response to increasing doses of methacholine (acetyl-β-methyl-choline chloride, Sigma-Aldrich) (0–25 mg/mL, 10% puff for 10 s) was used to determine lung function of dams on day 21 of lactation (day 42 of the experiment) as performed previously (Verheijden et al., 2014). Mice were anesthetized by intraperitoneal injection of ketamine (Vetoquinol S.A., France; 125 mg/kg) and medetomidine (Pfizer, Netherlands; 0.4 mg/kg) mixture. Data are expressed as average lung resistance (RL) in cm H2O/mL × sec^–1^.

#### Buxco

The Forced Pulmonary Maneuver System (Buxco Electronics Inc., Wilmington, NC, United States) was used to determine lung function of pups at 21 days of age as performed previously (Verheijden et al., 2014) (x). The baseline measurements were recorded and averaged for 3 min after 5 min acclimatization of the animals to the full body plethysmograph flow (FWBP). Bronchial hyperreactivity was assessed using the Penh (enhanced pause) values and the area under the curve (AUC) as the indexes.

### Blood Sampling and Serum Preparation

After measuring the lung function, dams and pups were euthanized using an overdose of pentobarbital by intra peritoneal injection (200 mg/kg; Nembutal^TM^, Ceva Santé Animale, Naaldwijk, Netherlands). Animals were terminated via cardiac puncture and blood samples collected. The blood was then coagulated for 30 min at room temperature, centrifuged at 8000 × *g* for 10 min, serum collected and stored at -20 until further analysis.

### Cotinine ELISA

Cotinine measurement was performed in serum samples using the mouse/rat cotinine ELISA kit (Calbiotech, CA, United States) according to the manufacturer’s instructions.

### Determination of Serum and Mammary Gland Homogenates EGF

Serum levels of EGF were determined by using a Mouse EGF DuoSet ELISA (R&D Systems, United States) according to the manufacturer’s instructions.

### Bronchoalveolar Lavage

After lung function measurement and immediately following euthanasia, a cannula was inserted into the trachea and lungs were lavaged four times with pre-warmed pyrogen-free saline (0.9% NaCl, 37°C) *in situ* (1 ml for dams and 0.5 ml for pups). Lungs were first lavaged with pyrogen-free saline (0.9%NaCl, 37°C) supplemented with protease inhibitor cocktail tablet (Complete Mini, Roche Diagnostics, Mannheim, Germany). This step was followed by three lavages with saline solution (0.9% NaCl, 37°C). The bronchioloalveolar lavage fluid was centrifuged at 4°C (400 × *g*, 5 min) and the supernatant of the first lavage was used for cytokine measurement. Cell pellets from the four lavages were pooled per animal, resuspended in 150 μl cold saline and counted under light microscopy using a Bürker-Türk chamber (magnification 100×). Differential BALF cell counts were performed on air-dried cytospin preparations stained with DiffQuik^TM^ (Merz & Dade A.G., Düdingen, Switzerland) and numbers of macrophages, neutrophils, eosinophils, and lymphocytes were identified according to standard morphology.

### *Ex vivo* Restimulation of Splenocytes

Fresh spleens were collected aseptically and homogenized using the top of a syringe to gently pass through a 70 μm nylon cell strainer. The collected cell suspension was washed and incubated with lysis buffer (Thermo Fisher, Netherlands), for 5 min to remove red blood cells. Splenocytes were washed and resuspended in RPMI 1640 medium (Lonza), supplemented with 10% heat-inactivated fetal bovine serum (FBS; Bodinco, Alkmaar, Netherlands), penicillin (100 U/mL)/streptomycin (100 μg/mL; Sigma-Aldrich) and β-mercaptoethanol (20 μM; Thermo Fisher Scientific). Total cell number was determined by using a Beckman Z1 coulter^®^ Particle Counter (Beckman, United States) and splenocytes (10^7^ cells/ml) were cultured in 96-well U-bottom plates (Greiner Bio-One B. V., Netherlands), either with medium or with 0.2 μg/mL anti-CD3 (Thermo Fisher, Netherlands). After 4 days stimulation, the supernatants were harvested and stored at -20°C until further analysis. The concentrations of MCP-1, IL-17A, IL-13, IL-22, and IFN-γ were measured using Bead-based immunoassays (Procartaplex, Thermo Fisher, Netherlands) according to the manufacturer’s instructions.

### Immunohistochemistry

The colon was immersed and fixed in fresh 10% neutral buffered formalin for at least 24 h and embedded in paraffin as a “Swiss roll” (Moran et al., 1983) to permit a complete microscopic examination. After paraffin embedding, 5 μm sections were cut. The sections were deparaffinized and endogenous peroxidase activity was blocked with 0.3% H2O2 (Merck, Darmstadt, Germany) in methanol for 30 min at room temperature and rehydrated in a graded ethanol series to PBS. For antigen retrieval, the slides were boiled in 10 mM citrate buffer (PH 6.0) for 10 min in a microwave. The slides were cooled down to room temperature, rinsed with PBS (3×) and blocked with 5% goat serum for 30 min at room temperature. Subsequently, sections were incubated with primary antibody rabbit anti-zonula occludens 1 (ZO-1) (Invitrogen) for 24 h. Thereafter, sections were washed and incubated with Alexa Fluor 568-conjugated anti-rabbit IgG (ZF-0511; Zhongshan Biotechnology Co., Ltd., Beijing, China; diluted at 1:50 in PBS with 1% BSA) for 1 h at RT. After washing, nuclear counterstaining was conducted using Hoechst (1:3000, Invitrogen) and sections were washed and mounted with ProLong Gold anti-fade reagent (Invitrogen). Immunofluorescence images were taken with Microscope Leica TCS SP8 X. Levels of laser intensity, confocal aperture, photomultiplier voltage, offset, scan speed, image size, filter, and magnification were equal for all images. Briefly, a minimum of four animals were selected per group and a minimum of four slides were stained for each animal. Afterward, confocal microscopic images were quantified for immunofluorescence intensity by ImageJ software (version: 1.52).

### Western Blot Analysis

Approximately, 50 mg of mammary gland specimens were lysed with 500 μl RIPA lysis buffer (Thermo scientific, Rockford, IL, United States) containing protease inhibitors (Roche Applied Science, Penzberg, Germany). Thereafter, total protein concentration was measured by a BCA protein assay kit (Thermo Scientific, Rockford, IL, United States). Standardized heat denaturized proteins were separated by electrophoresis (Criterion^TM^ Gel, 4–20% Tris–HCl, Bio-Rad Laboratories, Hercules, CA, United States) and transferred onto *Trans-*Blot Turbo Midi PVDF Transfer Packs (Bio-Rad Laboratories, Hercules, CA, United States). Membranes were blocked using PBS supplemented with 0.05% Tween-20 (PBST) and 5% milk proteins. Thereafter, membranes were incubated with primary antibodies for mouse anti-GAPDH (AM4300, Invitrogen, Carlsbad, CA, United States), mouse anti-E-cadherin (610182, eBioscience, San Diego, CA, United States) overnight at 4°C, followed by incubation with rabbit anti-mouse Immunoglobulins secondary antibody, HRP conjugated (P026002-2, agilent, United States). Blots were washed and developed with ECL reagents mix (Amersham Biosciences, Roosendaal, Netherlands) and the digital images were obtained using ChemiDoc^TM^ XRS + System (Bio-Rad Laboratories, Hercules, CA, United States). All the blots were exposed at the same time using the same exposure time. The intensity of the bands was quantified with Image Lab software (version 5.2, 2014, Bio-Rad Laboratories, Hercules, CA, United States) and normalized to anti-mouse GAPDH. Full uncut blots are included as Supplementary Data (Supplementary Figure 5).

### Statistical Analysis

Unless stated otherwise, results are expressed as mean ± standard error of mean (SEM). Data were statistically analyzed using a two-way ANOVA followed by a *post hoc* Bonferroni’s multiple comparisons test. A probability value *P* < 0.05 was considered significant. Statistical analyses were performed using GraphPad Prism software (version 6.04). The required sample size was calculated with G^∗^Power v3.1.9 based on a power of 80% and α = 0,05 and primary outcome parameter derived from previous observations of airway responsiveness which is the most relevant clinical parameter for CS-exposure.

## Results

### Effect of CS-Exposure on Pregnancy and Offspring

Smoke exposure in pregnant mice induced a reduction in breeding success compared to air- exposed animals (Table 1). Although the mating period was 4 days, and the exact first day of pregnancy is not known, data seems to indicate that prenatal CS-exposure significantly increases the length of pregnancy (Table 1). CS-exposure during pregnancy reduced the litter size by 12.6% compared to the air-exposed group (Table 1), however, this difference did not reach statistical significance. No significant difference in the sex ratio of pups was observed between air- and CS-exposed dams. Male and female pups from CS-exposed dams showed significantly lower body weight compared to pups from air-exposed dams. The spleen weight of pups from CS-exposed dams was also reduced compared to the spleen weight of pups from air-exposed dams. However, this was only significantly different between prenatally smoke- and air-exposed female pups (Table 1).

**TABLE 1:** Effect of CS-exposure on pregnancy and offspring.

Effect of CS smoke exposure on pregnancy and offspring		
	Experimental group	
	Air	CS
Duration of pregnancy^a^	21.53 ± 0.27	23 ± 0.33**
Litter size	4.66	4.07
No. of pregnant dams^b^	15	13
Female/male ratio	1.38	1.23
**Body weight (offspring)^c^**		
Female	10.56 ± 0.175	9.2 ± 0.249*
Male	10.56 ± 0.175	9.7 ± 0.3*
**Spleen weight/body weight ratio^d^**		
Female	0.0076 ± 0.0003	0.0062 ± 0.0003*
Male	0.0068 ± 0.0004	0.0058 ± 0.0002

*^*a*^Based on the first day of pairing (considered as gestational day 0), the mating period was 4 days.*

*^*b*^Values represent the mean (*n* = 30 animals/experimental group).*

*^*c*^Values represent the mean ± SEM (*n* = 10 animals/experimental group).*

^*d*^Values represent the mean ± SEM.

*Significantly different (*p* < 0.05) from the air-exposed group.

**Significantly different (*p* < 0.01) from the air-exposed group.

### Increased Cotinine Concentration in the Serum of Dams After Cigarette Smoke Exposure

Cotinine is the predominant metabolite of nicotine and is the most reliable biomarker to evaluate the level of smoke exposure. Cotinine concentrations were measured in the serum of air- and CS-exposed dams. Animals exposed to cigarette smoke during pregnancy and lactation showed a significantly higher cotinine level compared to air-exposed animals (Supplementary Figure 4).

### The AHR After Smoke Exposure Is More Obvious in Pregnant Dams Compared to Non-pregnant Dams

**FIGURE 1:**
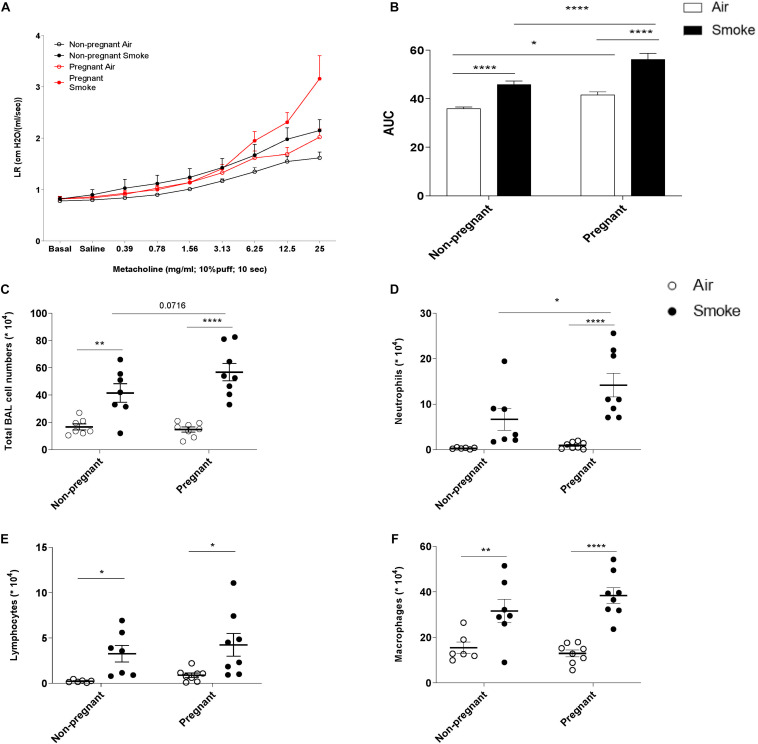
The increase in neutrophil numbers in BALF and AHR after smoke exposure is more obvious in pregnant dams compared to non-pregnant dams. Pregnant and non-pregnant animals were exposed to air/smoke during pregnancy and lactation and animals were sacrificed at the end of lactation. Lung resistance (RL) was measured after exposure to increasing doses of methacholine **(A)**. Areas-Under-Curve (AUC) were calculated for statistical analysis as mean ± SEM **(B)**. Lungs were lavaged and BALF was collected for total **(C)** and differential BAL cell counts, including neutrophils **(D)**, Lymphocytes **(E)**, and macrophages **(F)**. *P < 0.05, **P < 0.01, and ****P < 0.0001; as analyzed by two-way ANOVA followed by Bonferroni’s multiple comparisons test, *N* = 7 (Non-pregnant; air-exposed). *N* = 7 (Non-pregnant; CS-exposed). *N* = 10 (Pregnant; air-exposed). *N* = 10 (Pregnant; CS-exposed).

To study the effect of CS-exposure during pregnancy and lactation on lung function, the AHR to methacholine was evaluated one day after the last CS-exposure. Lung resistance (LR), as a measure for AHR, was found to be significantly higher due to CS exposure in both pregnant (P) and non-pregnant mice (NP) (Figure 1A). When CS-exposed pregnant animals were compared to non-pregnant animals, CS-exposure lead to a significant increased LR in pregnant animals. To increase our understanding of the smoke-induced effects on AHR, an area-under-curve (AUC) plot was generated, including all LR measurements (Figure 1B). AUC analysis indicates that CS significantly increased AHR in both non-pregnant and pregnant animals, while pregnant animals have significantly higher LR values compared to non-pregnant animals. Interestingly, pregnant animals were found to show higher AHR indicated by the significant difference between the air-exposed non-pregnant and pregnant animals.

### The Increase in Neutrophil Numbers in BALF After CS-Exposure Is More Obvious in Pregnant Dams Compared to Non-pregnant Dams

To investigate the extent of airway inflammation after CS-exposure, cell numbers in BALF were determined (Figures 1C–F). CS-exposure, but not air-exposure, leads to an increase in total BALF cell counts, as well as the in numbers of neutrophils, lymphocytes, and macrophages. Although, both pregnant and non-pregnant dams showed an increase in neutrophil infiltration after CS-exposure, the neutrophil influx was significantly higher in CS-exposed pregnant dams compared to CS-exposed non-pregnant dams.

### No Obvious Changes in BALF Cell Counts of Pups After Prenatal CS-Exposure

Pregnant and non-pregnant mice were exposed to either CS or air throughout pregnancy and lactation and adverse effects of prenatal CS-exposure on pulmonary function and inflammation were investigated in both female and male offspring. Due to the size of the pups, AHR was determined using the Forced Pulmonary Maneuver System as described in the method and materials and presented as an enhanced pause (Penh) value. AUC analysis indicates no significant difference between CS or air-exposed animals nor differences between the sexes (Supplementary Figure 2A). Upon BALF analysis, no significant differences were observed between prenatally CS- and air-exposed offspring in total cell counts, neutrophil, lymphocyte and macrophage numbers (Supplementary Figure 2B–E).

### Prenatal CS-Exposure Increases Cytokine Production After *ex vivo* Re-stimulation of Spleen Cells From the Offspring

**FIGURE 2:**
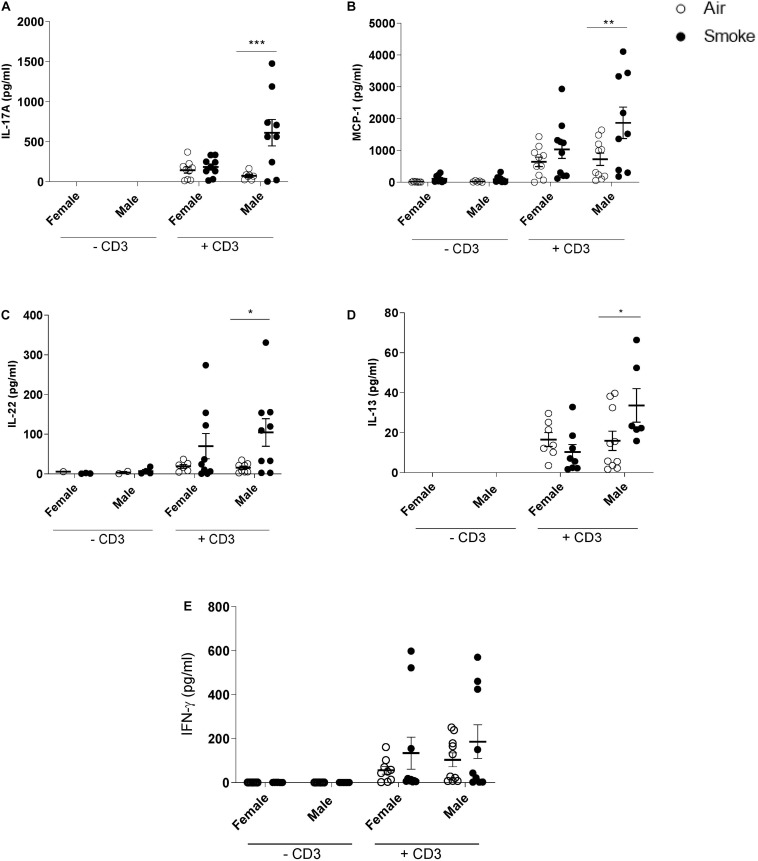
Prenatal CS-exposure increases cytokine production after *ex vivo* re-stimulation of spleen cells from the offspring. Prenatally air- and CS-exposed offspring were sacrificed at the end of lactation. **(A)** IL-17A, **(B)** MCP-1, **(C)** IL-22, **(D)** IL-13, and **(E)** IFN-γ concentrations were measured in supernatant after *ex vivo* stimulation of splenocytes with CD3 for 4 days (37°C, 5% CO2). Data are presented as mean ± SEM. *P < 0.05, **P < 0.01, ***P < 0.001, as analyzed by two-way ANOVA followed by Bonferroni’s multiple comparisons test (*n* = 8–10/group).

To investigate whether maternal CS-exposure leads to changes in immunocompetence in the offspring, spleen cell suspensions were *ex vivo* re-stimulated with anti-CD3 antibody to investigate T-cells responses (Figures 2A–E). In contrast to female offspring, maternal CS-exposure caused markedly elevated levels of IL-17A, MCP-1, IL-22, and IL-13 in anti-CD3 stimulated spleen cell culture supernatants of male pups. No significant differences were detected between male and female offspring of air-exposed dams (±CD3). IFN-γ levels seemed to be increased following anti-CD3 stimulation in both females and males, however, this did not reach significance. Notably, cytokine expression in unstimulated spleen cell culture supernatants were either very low or absent and no significance differences were observed between the male and female offspring.

### Effect of CS-Exposure on EGF Levels in Dams and Offspring

**FIGURE 3:**
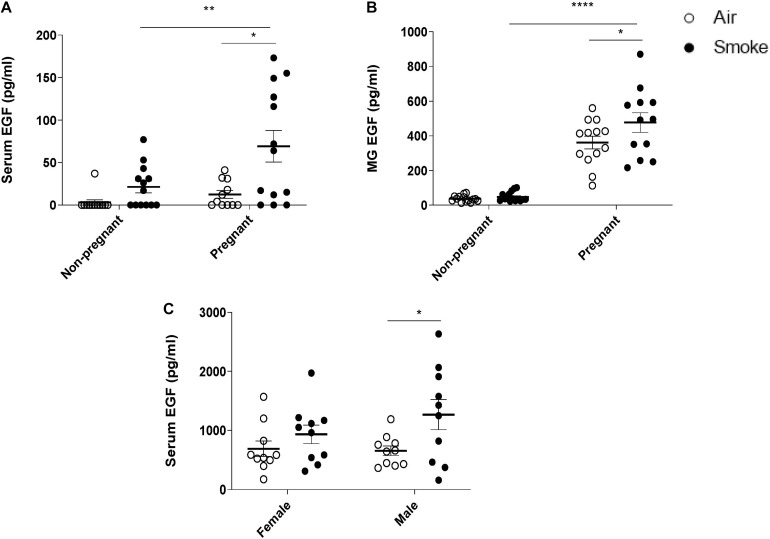
Effect of CS-exposure on EGF levels in dams and offspring. EGF concentrations were measured in **(A)** serum of dams, **(B)** mammary gland (MG) tissue homogenates, and **(C)** serum of offspring. Data are presented mean ± SEM. *P < 0.05, **P < 0.01, and, ****P < 0.0001, as analyzed by two-way ANOVA followed by Bonferroni’s multiple comparisons test (*n* = 10–15 mice/group).

CS-exposure increased serum EGF levels in both non-pregnant and pregnant females compared to air-exposed animals, however, only in pregnant females this increase was found to be significant. No differences in EGF levels were observed between pregnant and non-pregnant air-exposed dams (Figure 3A). The EGF concentration in the mammary gland tissue homogenates of CS-exposed pregnant dams was also significantly higher than air-exposed pregnant dams, while non-pregnant dams showed a very low EGF concentration in mammary gland tissue homogenates (Figure 3B). Non-pregnant dams did not show a significant increase in serum and mammary gland EGF levels after CS-exposure. Interestingly, prenatal CS-exposure resulted in an increase in serum EGF levels of the offspring. However, this effect only reached significance in the male pups (Figure 3C).

**FIGURE 4:**
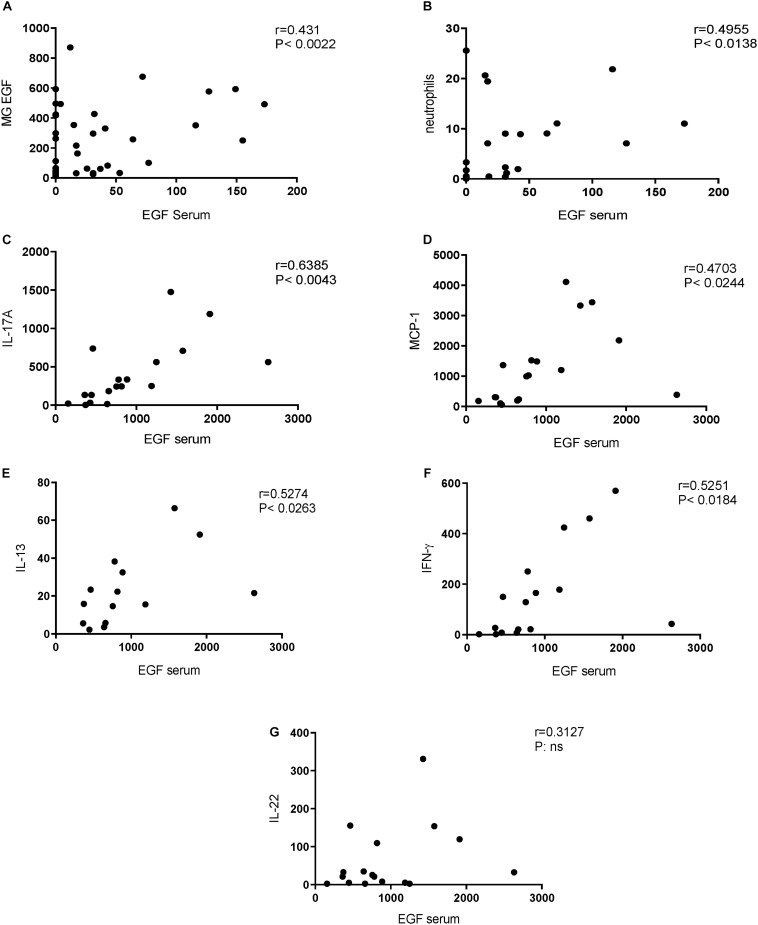
Positive correlations between EGF serum concentrations and neutrophil influx in BALF (dams) and cytokine release from CD3 stimulated splenocytes (offspring). Correlation of EGF serum concentration and EGF mammary gland (MG) concentration in dams **(A)**. Correlation of EGF serum concentration (pregnant and non-pregnant dams) and BALF neutrophil numbers in dams **(B)**. Correlation of EGF serum concentration in pups and IL-17A **(C)**, MCP-1 **(D)**, IL-13 **(E)**, IFN-γ **(F)**, and IL-22 **(G)**. Correlation was determined using the Spearman correlation test.

We hypothesized that pregnancy would induce dam EGF levels in the serum to the same extent as in the mammary glands. Indeed, a significant correlation was observed with higher serum levels correlating with higher EGF level of mammary gland tissue, depicted in Figure 4A. Next, it was investigated whether serum EGF levels could be related to inflammatory BALF cell numbers. Analyzing serum EGF levels and BALF neutrophil numbers of pregnant animals, a significant correlation was found (Figure 4B). In the offspring we hypothesized that serum EGF levels might impact on spleen cytokine production. In support of this hypothesis, a positive correlation between serum EGF levels and levels of IL-17A, MCP-1, IL-13, and IFN-γ, but not IL-22 in CD3 stimulated spleen supernatants was observed (Figures 4C–G, respectively).

### CS-Exposure During Pregnancy and Lactation Reduced E-Cadherin Protein Expression in Mammary Gland of Pregnant Dams Compared to Air-Exposed Pregnant Dams

**FIGURE 5:**
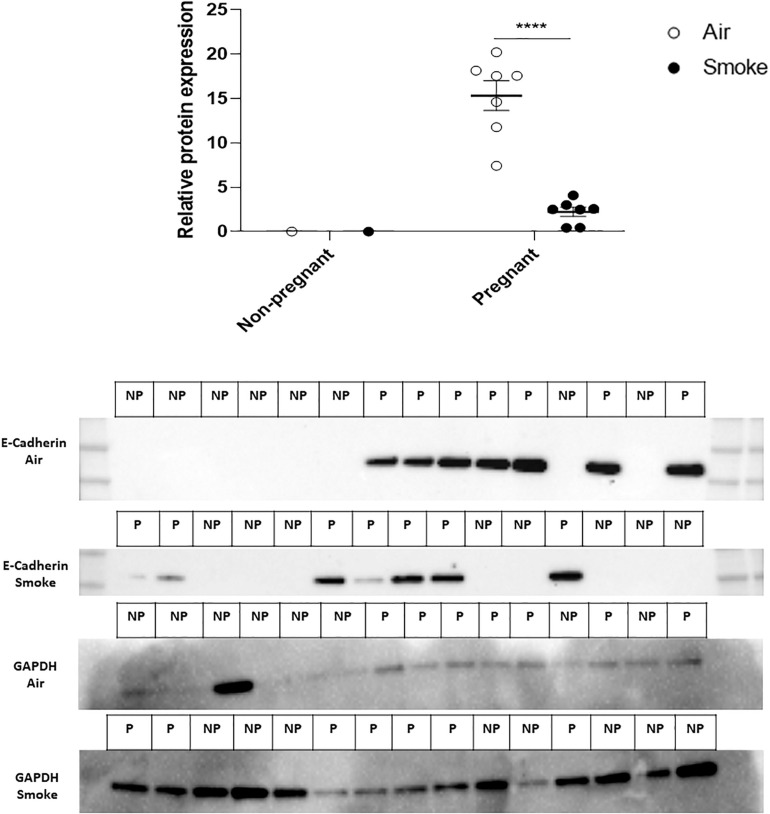
Effect of CS-exposure on E-cadherin expression in mammary gland tissue. Protein expression of E-cadherin in mammary gland tissue was measured by western blot analysis Expression of the protein was estimated by densitometry after normalization with GAPDH. Results are expressed as mean ratio (E-cadherin protein expression (OD/mm2) normalized to GAPDH) ± SEM (****P < 0.0001, as analyzed by two-way ANOVA followed by Bonferroni’s multiple comparisons test). (*n* = 7–8 mice/group).

To evaluate the effect of CS-exposure during pregnancy and lactation on the blood-milk barrier, protein expression of E-cadherin was measured in mammary gland tissue. Results indicated that the protein expression of E-cadherin was significantly reduced in mammary gland of CS-exposed pregnant dams compared to air-exposed dams (Figure 5). As expected, there was no E-cadherin protein expression in mammary gland tissue of non-pregnant dams.

## Discussion

Although cigarette smoking during pregnancy and lactation has been linked to several adverse pregnancy outcomes, smoking during pregnancy is still a prevalent behavior in many countries (Lange et al., 2018; Kondracki, 2019). In addition to active smoking, passive smoking (second hand smoke exposure) has been shown to have a high prevalence in low- and middle-income countries (Elf et al., 2018). Long-term direct and indirect CS-exposure (ETS, second hand smoking) causes a wide range of damaging health effects, including increased lung inflammation, protease activity, oxidant stress, and apoptosis (Gopal et al., 2016). To the best of our knowledge, there is no literature available examining the effect of maternal smoking on susceptibility to lung injury during pregnancy. Hence, this is the first article describing the adverse effects of maternal smoke exposure on AHR and the accumulation of inflammatory cells in the lungs of pregnant dams compared to non-pregnant mice. To have a level of exposure relevant to the human situation, we surveyed the literature for the correlation between cotinine levels and reported smoking behavior listed as light and heavy smokers (Jalili et al., 1998; Blackford et al., 2006; Xu et al., 2014). Unfortunately, the heterogeneity of reported cotinine levels and its association with smoking behavior between studies prevented us to qualify our reported cotinine levels (Supplementary Figure 4) as light or heavy smoking. However, based on TPM measurements, our total exposure is in line with previous similar *in vivo* experiments (Braber et al., 2012).

In the current study, non-pregnant mice exposed to CS showed a significant increase in the number of inflammatory cells in BALF, including neutrophils, lymphocytes, and macrophages, which is also observed by other authors (D’hulst et al., 2005; Martorana et al., 2005; Braber et al., 2010). Although the mean linear intercept (Lm), a measure of alveolar size/damage, was not determined in this 6-week study, other studies showed that cigarette smoke exposure for several weeks did not induce significant differences in Lm values in non-pregnant mice (Hartney et al., 2012; Rinaldi et al., 2012). Consistent with the findings observed in the BALF, airway responsiveness upon methacholine exposure in CS-exposed non-pregnant mice was significantly increased when compared to non-pregnant air-exposed mice. Interestingly, the CS-exposed pregnant animals showed a larger increase in AHR upon methacholine exposure compared to non-pregnant CS-exposed animals. The underlying mechanism on how CS exposure leads to increased AHR during pregnancy and lactation is not defined yet. However, several studies have indicated the link between neutrophil infiltration and AHR (Lukacs et al., 1996; Koga et al., 2013; Gao et al., 2017). AHR is a characteristic of many inflammatory lung diseases, and it has also been associated with chronic inflammation and infiltration of inflammatory cells, such as neutrophils (Vestbo and Hansen, 2001). Excessive infiltration and activation of neutrophils leads to the production and release of granule proteins, including serine proteases, matrix metalloproteinases, and myeloperoxidase. These factors contribute to the bronchial inflammation and induce structural changes, subsequent altered airway function and inflammatory processes via the induction of chemokine release by airway epithelial cells (Gernez et al., 2010; Koga et al., 2013; Victoni et al., 2016). Hence, a possible explanation for increased AHR is the link between pregnancy and higher neutrophil recruitment into the lungs. Pregnancy is associated with an increased number of white blood cells (Abbassi-Ghanavati et al., 2009). In healthy pregnant women, higher levels of peripheral blood granulocytes and a higher activation of peripheral blood leukocytes is detected compared with non-pregnant women (Sacks et al., 1998; Luppi et al., 2002; Harita et al., 2012). In addition, an elevated expression of surface adhesion molecules on granulocytes and granulocyte basal intracellular reactive oxygen species are observed in pregnant women. An *in vitro* study has highlighted the effect of pregnancy on neutrophil activation and neutrophil-endothelial interaction namely, exposure of neutrophils to conditioned medium from normal placental cultures stimulates neutrophil activation and neutrophil adhesion to endothelial cells (Wang et al., 2001). Enhancement of circulatory neutrophils and increased expression of surface adhesion markers, such as selectins, can lead to higher neutrophil recruitment into the lung. Here, we demonstrated, that maternal CS-exposure significantly increased the neutrophil number in the BALF of pregnant mice compared to non-pregnant animals. Overall, the findings from our study indicate that the response to CS-exposure in pregnant mice is different compared to non-pregnant mice as observed by a higher number of neutrophils in the BALF and increased AHR. This is an important finding and paves the way for future human studies to validate these observations.

Although various animal models have been used to investigate the association of neutrophil accumulation with the development of severe lung diseases, the underlying molecular mechanisms of neutrophil accumulation in the airways remain poorly understood. Several studies provided evidence that growth factors, such as EGF play an important role in neutrophil infiltration into the lung. CS induces EGF expression in ciliated cells of the airway epithelium and EGF up-regulation is observed in airway epithelium of smokers in association with airway dysfunction (Le Cras et al., 2010; Shaykhiev et al., 2013). We demonstrate that CS-exposed pregnant dams have significantly higher serum EGF levels compared to non-pregnant CS-exposed dams, and these higher levels positively correlate with neutrophil numbers in BALF of dams. EGF enhances the *ex vivo* production of chemotactic factors by the epithelial cells and its hypersecretion is associated with airway wall remodeling and lung inflammation (Shim et al., 2001; Hamilton et al., 2003; Holgate, 2011). An *in vitro* study, using the bronchial 16HBE cell line and primary bronchial epithelial cells from healthy individuals, indicated that exposure to EGF enhances neutrophil accumulation via stimulation of pro-inflammatory cytokines and chemokines (Uddin et al., 2013). A clinical study showed that EGF induces a significant up-regulation of pro-neutrophilic factors, like IL-6, IL-8, GM-CSF, and TNF-α in asthmatic patients. Overall, this would suggest that high serum EGF levels during pregnancy potentially sensitize an individual to environmental-induced lung injury mediated through increased accumulation and activation of innate cells. Besides EGF, several studies have shown that hormonal fluctuations could also lead to significant changes in lung function, including AHR (Carey et al., 2007; Card and Zeldin, 2009). For instance, estrogen receptor-α knockout mice exhibited lung function abnormalities and increased airway responsiveness to inhaled methacholine under basal conditions (Carey et al., 2007). However, these findings await clinical validation where the relation between EGF and susceptibility to lung injury needs to be further researched.

Apart from these effects of CS-exposure on dams, results from different *in vivo* studies indicate that prenatal CS-exposure can also lead to altered immune responses in the offspring. In the present study, we observed that prenatal CS-exposure leads to markedly elevated IL-13, IL-22, and IL-17A in anti-CD3 stimulated spleen cell culture supernatants of 3-week-old male pups. These cytokines play an influential role in inducing allergic reactions. For instance, prenatal allergen-specific Th-2 responses, particularly IL-13 responses, have been associated with atopic risk or subsequent allergic diseases (Gabrielsson et al., 2001; Kopp et al., 2001; Spinozzi et al., 2001). Moreover, it has been reported that serum levels of IL-22 are higher in patients with severe asthma than those seen healthy control subjects (Zhao et al., 2010). The pro-inflammatory properties of IL-22 are demonstrated as IL-22 is detected at the sites of allergic airway inflammation (Takahashi et al., 2011). IL-17 plays a critical role in allergic responses and autoimmune inflammation (Wang and Liu, 2008; Eun-Hyung Lee et al., 2010; Robinson et al., 2013). The increased level of the IL-17 and IL-22 directly correlates with increased disease severity (i.e., increased AHR). We further showed that prenatal smoke exposure significantly increases the MCP-1 secretion by splenocytes followed by anti-CD3 stimulation. MCP-1, as monocyte chemoattractant protein, is associated with the development of polarized Th2 responses (Deshmane et al., 2009). Additionally, MCP-1 induces polarization of Th0 cells toward a Th2 phenotype (Gu et al., 2000). Overall, the increased level of all cytokines/chemokines released by anti-CD3 stimulated splenocytes of prenatal CS-exposed male pups might be indicators for a predisposition toward Th2 responses. Indeed, prenatal CS-exposure has been linked with augmented Th2 differentiation along with enhanced Th2-type cytokine production, a known contributor to allergic inflammation (Murata et al., 2002; Noakes et al., 2003). Some studies have revealed that CS-exposure during pregnancy will raise the risk of asthma in the offspring (Zacharasiewicz, 2016; Huang et al., 2017). Huang et al. (2017) reported that early life CS-exposure aggravates OVA-induced asthma, airway inflammation, and increased infiltration of neutrophils, eosinophils, and other inflammatory cells.

Growth factors affect a wide variety of physiological processes including cell proliferation, differentiation and immunological responses. However, the abnormal production or regulation of growth factors can lead to development of various diseases, such as cancers and bronchopulmonary dysplasia (Sporn and Roberts, 1986; Thébaud and Abman, 2007). EGF is absent from commercially available infant formulas and preclinical data indicate that supplementation of formula with EGF supports growth, maturation and function of the gastroenteral tract (GI) (O’Loughlin et al., 1985; Berseth, 1987; Opleta-Madsen et al., 1991). The epidermal growth factor family has been known for its ability to stimulate cell proliferation and deregulation of the members of EGF family and their receptors has been shown to be closely associated with inflammatory diseases, such as atherosclerosis (Zeboudj et al., 2018). We observed that the concentration of EGF in the mammary gland tissue homogenates of CS-exposed pregnant dams was significantly higher than air-exposed pregnant dams and this increase was positively correlated with serum EGF levels. Moreover, prenatally exposed pups also demonstrated higher serum EGF levels. Blood-milk barrier disruption would be a candidate for factors eliciting higher EGF levels in mammary gland tissue homogenates. Supporting this, our results indicate that maternal smoke exposure reduced the E-cadherin expression in the mammary gland tissue, which is known as an adherence junction protein. E-cadherin has been identified as a primary classical cadherin in luminal epithelial cells and plays a crucial role in mammary gland differentiation and cell survival of alveoli in the lactating gland (Boussadia et al., 2002; Shamir and Ewald, 2015). Boussadia et al. (2002) showed that E-cadherin mutant mouse were not able to lactate and the differentiation of the mammary alveolar epithelium was affected in these mice. Several studies have noted the role of CS exposure in the downregulation of adherence junctions in lung tissue (Xi et al., 2000; Di Vincenzo et al., 2019). An *in vitro* study using an air-liquid interface system with human primary airway epithelial cells showed that smoke exposure is associated with reduced E-cadherin expression (Nishida et al., 2017). Besides the local effect of smoke exposure, results from different *in vivo* studies indicate that CS can affect other organs as well. Interestingly, a mouse model of CS-exposure presented a disturbed intestinal integrity as observed by reduction in ZO-1 expression in the small intestine (Zuo et al., 2014). In addition, CS-exposure causes intestinal barrier dysfunction of the small bowel with increased intestinal permeability and bacterial translocation in a murine smoke exposure model (Zuo et al., 2014). These effects might contribute to the increased serum EGF level observed in our prenatally CS-exposed pups. In addition, using an immunofluorescent staining, we found that there is a tendency toward a reduction in ZO-1 expression in the colon tissue of parentally CS-exposed male pups (Supplementary Figure 3). This suggest that CS exposure can exert several deleterious effects on the intestinal barrier, which might result in higher absorption of EGF into the circulation. In the current study, we have demonstrated a strong positive correlation between the serum EGF levels and IL-17A, MCP-1, IL-13, IFN-γ levels in anti-CD3 stimulated spleen cell culture supernatants of 3-week-old male pups. EGF promotes cell growth and differentiation by binding to its receptor, the epidermal growth factor receptor (EGFR) EGFR (Harris et al., 2003; Dvorak, 2010). Binding of EGF to the EGFR extracellular domain (EGFR-ECD) promotes its inactive-to-active conformational transition (activation), but the relevant detailed mechanism remains still elusive. EGFR is a transmembrane protein, which controls major signaling pathways, including cell survival, proliferation, and migration. Human and experimental studies have shown that an increased expression of EGFR by autocrine ligand stimulation leads to dysregulation of the downstream signaling system, which consequently results in a range of pathophysiological disorders such as cancer development (Zeboudj et al., 2018). Splenic CD4 + T cells and human blood T cells express EGFR, especially in response to CD3 (Zeboudj et al., 2018). EGFR signaling is crucial in CD4 + T cell homeostasis in both humans and mice and this role was further proved by epidemiological findings where patients treated with EGFR inhibitors might be particularly susceptible to infections (Burtness et al., 2012; Minutti et al., 2017). Moreover, inhibition of EGFR leads to a significant reduction of *in vitro* and *in vivo* cell proliferation and Th1/Th2/Th17 cytokine production. Moreover, smoke exposure induces EGFR phosphorylation and activation in different tissues (Filosto et al., 2012), which consequently leads to overproduction of cytokines (Paul et al., 2014). The strong correlation between EGF levels and cytokines measured in the spleen cultures strongly indicate a role for EGF as an immunomodulator. However, other than EGF, components such as nicotine have also been shown to have immunomodulating properties and capable of reducing mitogen-induced splenic T-cell proliferation (Kalra et al., 2004). It is of relevance to study a role for nicotine in these effects because nicotine has been found in the breastmilk of smoking mothers and cotinine could be detected in the urine of infants born to smoking mothers, suggesting systemic availability of nicotine and nicotine metabolites (Mascola et al., 1998; Amir, 2001). In support of our findings, it was previously shown that activation of EGF by EGF-specific ligands increased mast cell chemotactic protein transcription by dental follicle cells, indicating direct immune effects following EGF activation (Liu et al., 2008). It is of relevance to study the contribution of EGF in relation nicotine for their effect on splenic restimulations to further delineate a possible EGF specific effect. Future studies should then include naive mouse splenic restimulations in the absence or presence of nicotine, nicotine metabolites or EGF.

Previous studies have highlighted the role of sex difference in adaptive immune responses. For instance, it has been shown that male rats (early in life) have a larger thymus and a higher thymocyte count compared to female rats (Leposavić et al., 1996; Leposavić et al., 2011). In addition, clinical studies have demonstrated that natural killer cell frequencies are higher in male compared to female children (Lee et al., 1996). In the current study, prenatally CS-exposed male offspring showed higher immune responses to CD3 stimulation compared to female animals. This finding is in agreement with previous data of Casimir et al. (2010), who found that human infant males have higher pro-inflammatory responses compared to females following stimulation with LPS. The findings that male offspring are more vulnerable than the female to maternal smoke exposure is supported by recent studies (Stevenson et al., 2000; Kirchengast and Hartmann, 2009; Murphy et al., 2012; Miyake et al., 2013; Roy et al., 2014; Owili et al., 2018). Other studies on different outcomes also indicate that males are more susceptible to develop insulin resistance, obesity, adverse lung outcomes, and dyslipidemia (Shahid et al., 2008; Ng et al., 2009; Wang et al., 2020).

Despite the known detrimental effect of smoke exposure during pregnancy and lactation, in the European Union, 10–27% of the pregnant women who smoke continue smoking during pregnancy. This study is the first to indicate that mothers who smoke during pregnancy and lactation may be at greater risk of developing lung injuries. We showed that CS-exposure during pregnancy and lactation significantly increases the airway hyperresponsiveness and neutrophil infiltration into lung of dams compared to non-pregnant dams. In addition, we have demonstrated that prenatal and postnatal CS-exposure alters the immune responses in the offspring. Spleen cells isolated from prenatally CS-exposed animals showed higher sensitivity to CD3 stimulation compared to the air-exposed group. Moreover, in the current study we have indicated that EGF might be a possible missing link in maternal CS-exposure and infant’s immune hypersensitivity. However, further research is needed to elucidate the mechanisms by which prenatal and postnatal CS-exposure leads to higher immune responses.

## Data Availability Statement

The original contributions presented in the study are included in the article/ Supplementary Material, further inquiries can be directed to the corresponding author.

## Ethics Statement

The animal study was reviewed and approved by Dierexperimentencommissie Utrecht.

## Author Contributions

SB, HJ, JB, JG, and GF designed research. HJ, MD, TL-M, and IA performed research. HJ and TL-M analyzed data. HJ, GF, JB, and SB wrote the manuscript. All authors contributed to the article and approved the submitted version.

## Conflict of Interest

JG and JB are employees of Danone Nutricia Research. The remaining authors declare that the research was conducted in the absence of any commercial or financial relationships that could be construed as a potential conflict of interest.

## Publisher’s Note

All claims expressed in this article are solely those of the authors and do not necessarily represent those of their affiliated organizations, or those of the publisher, the editors and the reviewers. Any product that may be evaluated in this article, or claim that may be made by its manufacturer, is not guaranteed or endorsed by the publisher.
