# Supplementary material for: The Effects of Maternal Smoking on Pregnancy and Offspring: Possible Role for EGF?
Source: Front Cell Dev Biol. 2021 Aug 16;9:680902. doi: 10.3389/fcell.2021.680902 (PMC8415274; doi:10.3389/fcell.2021.680902)
Supplement: Supplementary file 1 [file Data_Sheet_1.docx]

Supplementary Material

# Supplementary Figures


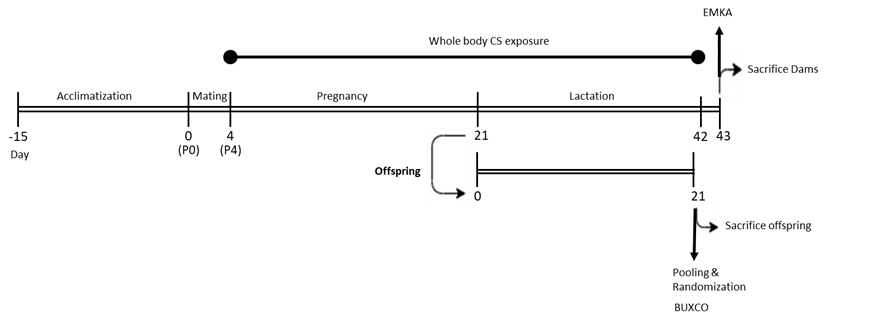


Fig. S1. Experimental design

Dams were exposed to either air or diluted mainstream cigarette smoke from the reference cigarettes 1R6F in whole-body chambers from pregnancy day four until the end of lactation. Exposures were performed for 45 min/day, 7 days/week for 6 weeks. Pups were sacrificed at lactation day 21 and dams were sacrificed at lactation day 22 (day 43 of the experiment).


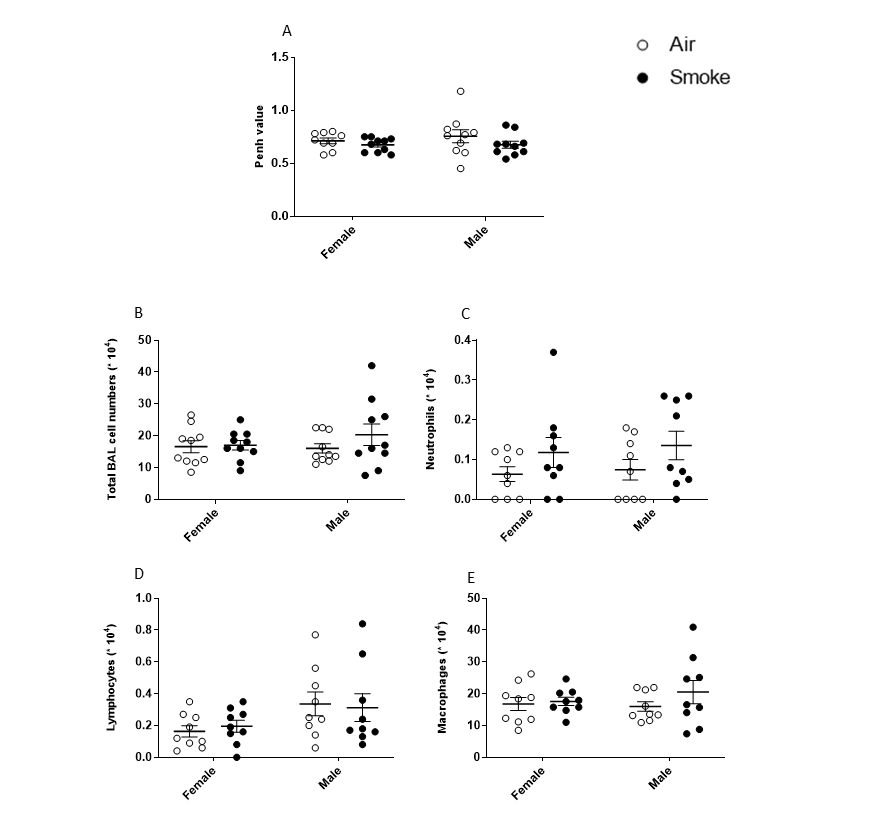


**Fig. S2. No obvious changes in BALF cell counts of pups after prenatal CS exposure**

Airway hyperresponsiveness in pups was determined using the Forced Pulmonary Maneuver System at lactation day 21 and was presented as an enhanced pause (Penh) value as described in materials and methods (A). Lungs were lavaged and BALF was collected for total (B) and differential cell counts, including neutrophils (C), lymphocytes (D) and macrophages (E). Values are expressed as mean (x10^4^) ± SEM. n = 8-10 mice/group.


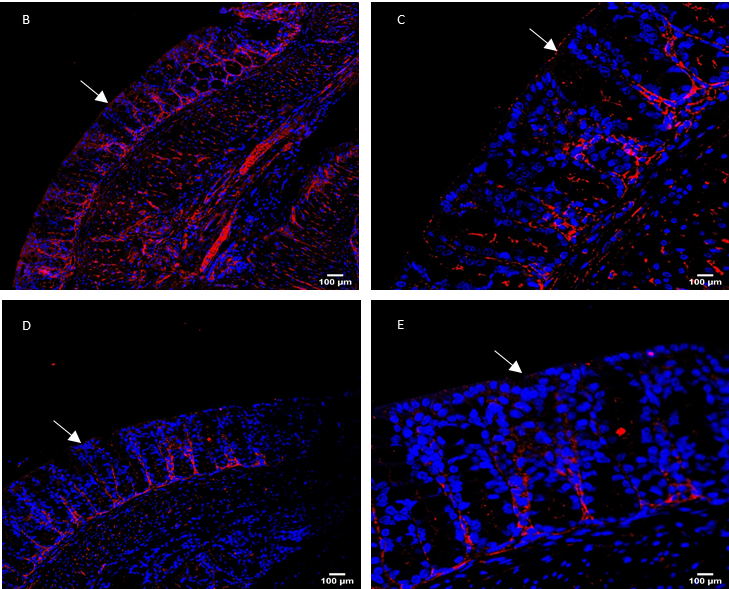


A

**Fig. S3. ZO-1 expression in colon after prenatal cigarette smoke exposure.**

ZO-1 expression was detected and quantified in swiss-rolled paraffin sections obtained from colon tissue of prenatally air- and smoke-exposed pups by using immunofluorescence microscopy as described in materials and methods. (A) Quantitative analysis of the immunofluorescence intensity of ZO-1. (B, C) Representative images of prenatally air- and (D, E) smoke-exposed pups. Magnification 100X (B, D) and 200X (C, E). Results are expressed as mean ± SEM; as analyzed by t-test.


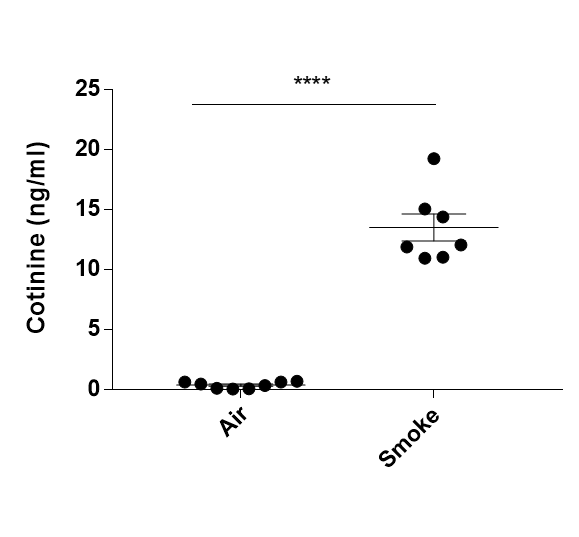


**Fig S4. Increased cotinine levels after cigarette smoke exposure.**

The cotinine levels were measured in serum of dams exposed to air or cigarette smoke. Results are expressed as mean ± SEM. ****P < 0.0001; as analyzed by t-test.


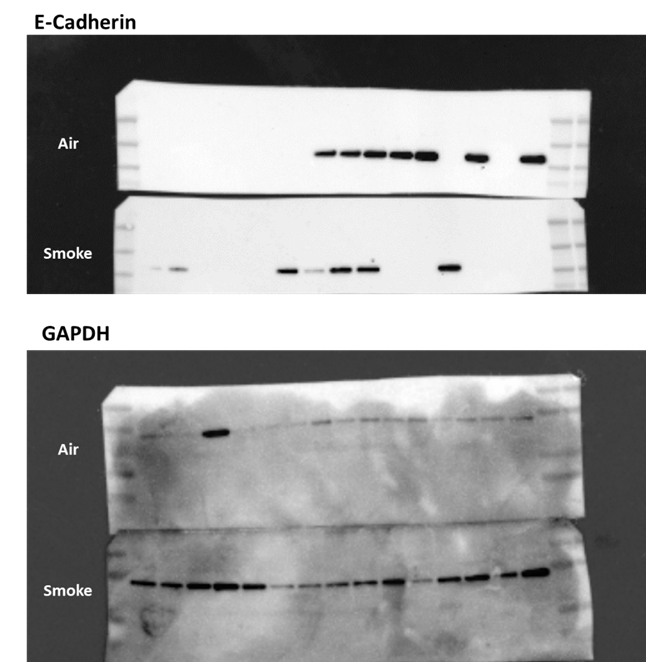


**Fig S5. Effect of CS-exposure on E-cadherin expression in mammary gland tissue.**

Protein expression of E-cadherin in mammary gland tissue was measured by western blot analysis (Full un-cut blots).
